# Visualization of both proximal M2-MCA segments in patients (the Tilted-V Sign) with acute M1-MCA occlusion stroke is associated with better procedural and prognostic outcomes

**DOI:** 10.3389/fneur.2022.1041585

**Published:** 2022-12-13

**Authors:** Amit Azriel, Anat Horev, Elad Avraham, Farouq Alguayn, Yair Zlotnik, Gal Ifergane, Yuval Zeev Sufaro, Yotam Dizitzer, Israel Melamed, Ilan Shelef, José E. Cohen, Ronen R. Leker, Asaf Honig

**Affiliations:** ^1^Department of Neurosurgery, Soroka University Medical Center and Faculty of Health Sciences, Ben-Gurion University of the Negev, Beer Sheva, Israel; ^2^Department of Neurology, Soroka University Medical Center and Faculty of Health Sciences, Ben-Gurion University of the Negev, Beer Sheva, Israel; ^3^Department of Research Center, Soroka University Medical Center, Beer Sheva, Israel; ^4^Department of Radiology, Soroka University Medical Center and Faculty of Health Sciences, Ben-Gurion University of the Negev, Beer Sheva, Israel; ^5^Department of Neurosurgery, Hadassah-Hebrew University Medical Center, Jerusalem, Israel; ^6^Department of Neurology, Hadassah-Hebrew University Medical Center, Jerusalem, Israel

**Keywords:** acute ischemic stroke, collateral cerebral circulation, endovascular thrombectomy, large vessel occlusion, Tilted-V Sign

## Abstract

**Introduction:**

We aimed to assess the clinical significance of M1-MCA occlusion with visualization of both MCA-M2 segments [“Tilted-V sign” (TVS)] on initial CT angiography (CTA) in patients with acute ischemic stroke (AIS) undergoing endovascular thrombectomy (EVT).

**Methods:**

Data for patients with consecutive AIS undergoing EVT for large vessel occlusion (LVO) in two academic centers are recorded in ongoing databases. Patients who underwent EVT for M1-MCA occlusions ≤ 6 h from symptom onset were included in this retrospective analysis.

**Results:**

A total of 346 patients met the inclusion criteria; 189 (55%) had positive TVS. Patients with positive TVS were younger (68 ± 14 vs. 71 ± 14 years, *P* = 0.028), with similar rates of vascular risk factors and baseline modified Rankin scores (mRS) 0–2. The rates of achieving thrombolysis in cerebral ischemia (TICI) 2b-3 were similar to the two groups (79%), although successful first-pass recanalization was more common with TVS (64 vs. 36%, *p* = 0.01). On multivariate analysis, higher collateral score [odds ratio (OR) 1.38 per unit increase, *p* = 0.008] and lower age (OR 0.98 per year increase, *p* = 0.046) were significant predictors of TVS. Patients with positive TVS had higher post-procedural Alberta Stroke Program Early CT Score (ASPECTS; 6.9 ± 2.2 vs. 5.2 ± 2.3, *p* = 0.001), were discharged with lower National Institutes of Health Stroke Score (NIHSS; 6±6 vs. 9±7, *p* = 0.003) and higher rates of mRS 0–2 (29.5 vs. 12%, *p* = 0.001), and had lower rates of 90-day mortality (13.2 vs. 21.6%, *p* = 0.038). However, TVS was not an independent predictor of functional independence (OR 2.51; 95% CI 0.7–8.3).

**Conclusion:**

Tilted-V Sign, an easily identifiable radiological marker, is associated with fewer recanalization attempts, better functional outcomes, and reduced mortality.

## Introduction

**Figure 1:**
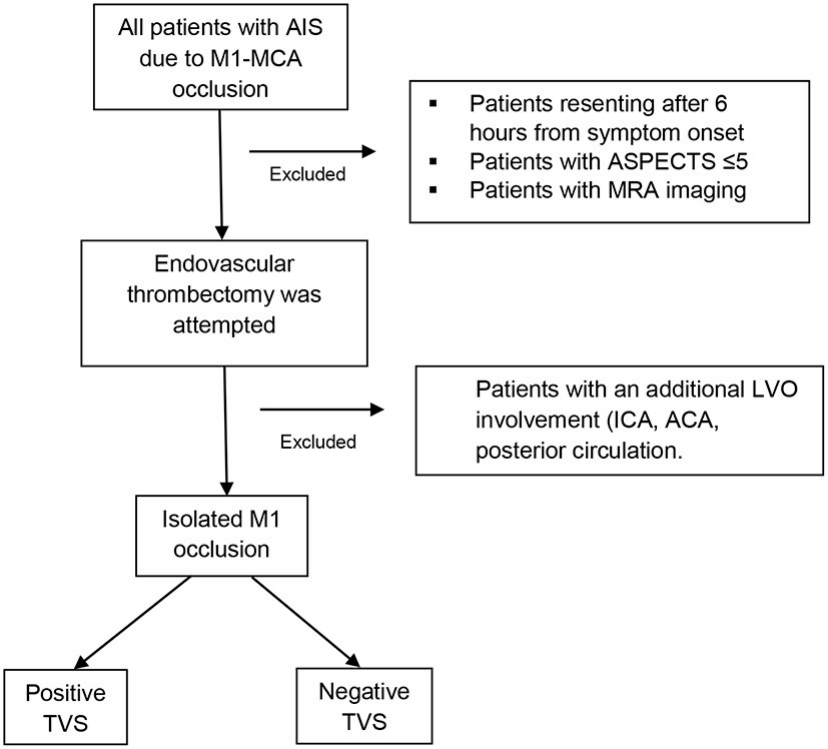
Flowchart of patient inclusion. AIS, acute ischemic stroke; ED, emergency department; EVT, endovascular treatment; ICA, internal carotid artery; ACA, anterior cerebral artery.

Outcomes in acute ischemic stroke (AIS) patients with large artery occlusions in the anterior circulation who are not treated with reperfusion therapies are extremely poor, notably in the terminal internal carotid artery (ICA) and proximal middle cerebral artery (MCA) occlusions (1, 2). Recanalization is the goal of medical and endovascular treatment (EVT) for acute proximal large vessel occlusion (LVO) to reduce penumbra loss (3, 4). Many factors affect the success of recanalization, including clot burden and composition, treatment strategy (i.e., systemic thrombolysis vs. EVT), site of clot impaction, and collateral supply (5–9). Reliable imaging data can possibly provide information regarding subgroups of patients who should either be treated with a specific recanalization technique or will have a good functional outcome when treated in an extended time window (6–24 h from symptom onset). Commonly used pretreatment neuroradiological assessment tools include the Alberta Stroke Program Early CT Score (ASPECTS) on the initial non-contrast CT scan (NCCT), the collateral score, and the clot burden score (10–13). To the best of our knowledge, the presence of an M1-MCA occlusion with visualization of both MCA M2 segments on CTA was not previously discussed in the literature. We defined this specific pattern of occlusion as the Tilted-V sign (TVS) (Figure 1) and aimed to assess it as a potential imaging marker for the outcome.

## Methods

### Study population

Data for patients with consecutive AIS who underwent EVT for LVO in the two participating academic centers were recorded in ongoing databases. The prospective registry included baseline demographics and clinical characteristics, the National Institute of Health Stroke Scale (NIHSS), and modified Rankin score (mRS) at admission and discharge and imaging data. Both institutions have similar patient selection protocols for EVT, and patients with M1 occlusions and ASPECTS ≥6 presenting within 6 h from symptom onset are generally sent for EVT. Post-procedure treatment protocols are also similar, with an emphasis on maintaining systolic blood pressure (BP) <140 mmHg in patients with modified Thrombolysis in Cerebral Infarction (mTICI) scores of 2b-3, which is considered to be successful target vessel recanalization. Unless there was a contraindication, the preferred vascular imaging upon arrival was CTA from the arch of the aorta to the vertex of the head. CTA included two phases, arterial and venous, to identify and rule out patients with subocclusive M1 thrombosis. Whenever using iodine contrast was contraindicated, an magnetic resonance angiography (MRA) was performed with additional fluid-attenuated inversion recovery (FLAIR), diffusion-weighted imaging (DWI), and susceptibility-weighted imaging (SWI) sequences.

For the current study, a retrospective analysis of patients from the registries over the 5 years from 2014 to 2018 was identified. Patients aged ≥ 18 years who underwent EVT for M1-MCA occlusions within 6 h from symptom onset, with or without IV thrombolysis (IVT) prior to EVT, were included in the study. Those with LVO in additional territory apart from the MCA, including the ICA and anterior or posterior cerebral arteries, were excluded to enable stronger comparisons of characteristics and outcomes for patients with solely M1-MCA LVO (Figure 1). As we aimed to assess the clinical significance of the TVS on CTA, patients who underwent MRA upon arrival were also excluded.

### Imaging data

**Figure 2:**
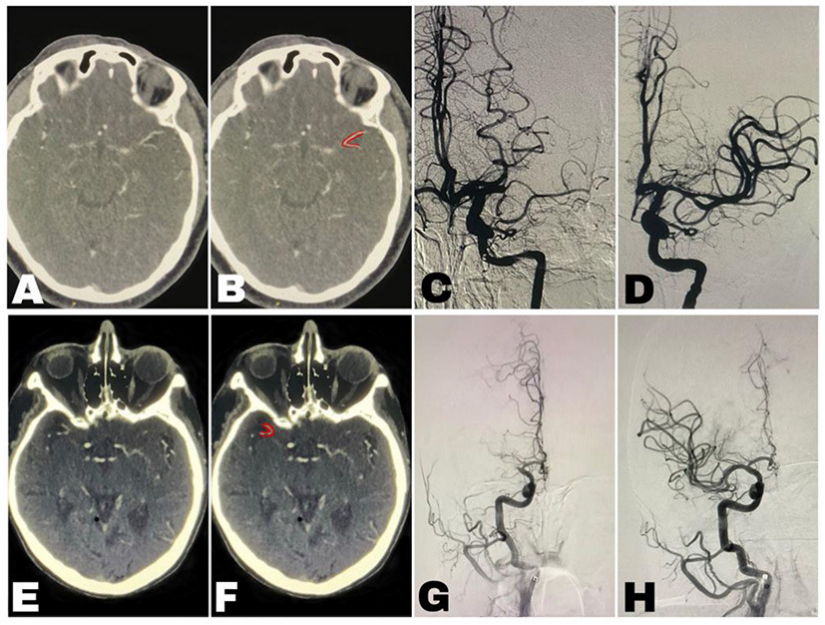
Imaging of two patients [**(A–D)** patient-1, **(E–H)** patient-2]. **(A,E)** The “Tilted-V Sign” unmarked. **(B,F)** The “Tilted-V Sign” (marked in red)—axial images of CT angiogram of the head showing filling of both proximal M2 segments of the Middle Cerebral Artery (MCA) distal to an occluded, not visualized M1-MCA segment, thereby creating a Tilted-V shape. **(C,G)** Digital subtraction angiography (DSA) before endovascular thrombectomy (EVT) showing complete occlusion of the M1-MCA segment but without visualization of vessels distal to the occlusion. **(D,H)** DSA following EVT showing successful recanalization.

**Figure 3:**
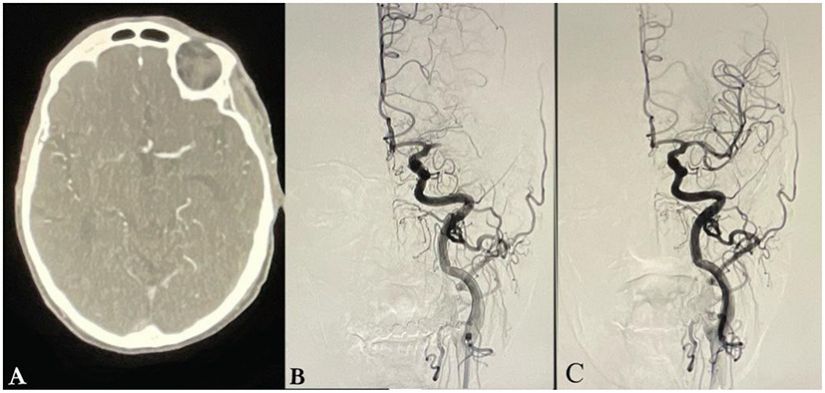
Imaging of a patient without a “Tilted-V Sign.” **(A)** Axial images of CT angiogram of head showing lack of flow beyond the occluded M1-Middle Cerebral Artery (MCA) segment without filling of proximal M2 segments of the middle cerebral artery (MCA). **(B)** Digital subtraction angiography (DSA) before endovascular thrombectomy (EVT) showing complete occlusion of the M1-MCA segment but without visualization of vessels distal to the occlusion. **(C)** DSA following EVT showing successful recanalization.

Imaging data retrieved from the institutional registries included pre-procedural NCCT and CTA, and post-procedural NCCT and digital subtraction angiography (DSA). All imaging data were reviewed by senior stroke neurologists blinded to the clinical information (AH and RRL). They verified the occlusion of the M1-MCA segment in both the early and delayed phases of CTA. They excluded patients with additional vessel occlusion. They evaluated the presence of filling of both M2 segments distal to the suspected occlusion on axial slices of CTA images (Figure 2). When this pattern was identified on the initial CTA, we termed it the Tilted-V sign (TVS). Complete M1-MCA occlusion had to be verified in the DSA in addition to the admission CTA. Patients with an M1-MCA occlusion with visualization of only one of the M2 on the initial CTA were regarded as TVS negative (Figure 3).

The collateral grading system was scored on a scale of 0–3 using the initial phase of the CTA (5). A score of zero indicated absent collateral supply to the occluded MCA territory. A score of 1 indicated collateral supply filling ≤ 50% but >0% of the occluded MCA territory. A score of 2 was given for collateral supply filling >50% but <100% of the occluded MCA territory. A score of 3 was given for 100% collateral supply of the occluded MCA territory.

### Endovascular treatment

Digital subtraction angiography and EVT were undertaken on a biplane angiography system (Allura Xper, Philips, Eindhoven, The Netherlands). For the intervention, patients were either under general anesthesia or conscious sedation. Three possible techniques were used for EVT: (1) aspiration thrombectomy (Penumbra System; Penumbra Inc, Alameda, CA), (2) mechanical thrombectomy with stent retriever (Solitaire FR, ev3 Neurovascular, Irvine, CA), or (3) combined technique using both stent retriever and aspiration. All procedures were performed according to the preferences and the discretion of the treating endovascular physician. EVT was halted when successful reperfusion, defined as mTICI 2b or 3, was achieved, or if reperfusion could not be achieved within 2 h from groin puncture. Time from groin puncture to successful recanalization was prospectively recorded in the institutional databases.

### Prognostic evaluation

The outcome was assessed by the treating physician using the mRS upon discharge from the Neurology Department, regardless of the patient's destination. Discharge from the Neurology Department at both medical centers took place within 2 weeks of admission. mRS 0–2 upon discharge was regarded as a good functional outcome. We do not have a full clinical evaluation on all patients at 90-day follow-up, and therefore we excluded this variable from the analysis. However, the computerized system was constantly updated in case of death, thus allowing us to accurately report on 90-day mortality.

### Statistical analysis

All statistical analyses were performed using the SPSS software version 24.0 (IBM, Armonk, NY, USA). Descriptive statistics were reported for all variables as means and standard deviations for numerical variables and as percentages for categorical data. For categorical variables, *P*-values were calculated by the chi-square test or the phi coefficient when appropriate. Means were compared by the one-sample *t*-test or the Mann–Whitney *U*-test when appropriate. A *p* ≤ 0.05 was considered statistically significant. Multivariate progressive regression models controlling for all variables that yielded a statistically significant *p*-value were used to control for possible confounders.

### Ethical considerations

The study was approved by the medical institution's review boards (0149-2015 and HMO-0378-2018). Due to the retrospective nature of the study, informed consent was waived. Data were anonymized.

## Results

### Baseline characteristics

A total of 346 patients with M1-MCA occlusions who met inclusion criteria were evaluated, including 189 (55%) with positive TVS (Table 1). Patients with positive TVS were younger (68 ± 14 years vs. 71 ± 14 years, *P* = 0.028) and had a slightly better baseline mRS (0.9 ± 1.4 vs. 1.3 ± 1.6, *p* = 0.03) compared to patients without TVS. However, rates of baseline mRS > 2 (36 vs. 37%, *p* = 0.2) and comorbidities were similar between the two groups. Admission NIHSS also did not differ (15 ± 7 vs. 16 ± 7, *p* = 0.062). Mean ASPECT and collateral scores on baseline imaging were mildly higher in the positive-TVS group (8.3 ± 1.2 vs. 7.5 ± 1.6, *p* = 0.02 and 2.1 ± 0.7 vs. 1.7 ± 0.8, *p* = 0.04, respectively). In a multivariate analysis, higher collateral score [odds ratio (OR) 1.38 per unit increase, 95% CI 1.08–1.68, *p* = 0.008] and lower age (OR 0.98 per year increase, 95% CI 0.96–0.99, *p* = 0.046) were significant predictors of TVS (Table 2).

**Table 1:** Baseline characteristics of patients with and without TVS.

**Variable**	**Positive TVS** ***N* = 189 (55%)**	**Negative TLS** ***N* = 157 (45%)**	**Total** ***N* = 346 (100%)**	***p*-value**
Age	68 ± 14	71 ± 14	69 ± 14	**0.028**
Sex (male)	78 (41.2%)	67 (42.6%)	145 (44%)	0.774
AF	83 (44%)	78 (50%)	161 (46.5%)	0.327
HTN	119 (63%)	110 (70%)	229 (66%)	0.185
DM	61 (32%)	50 (32%)	111 (32%)	0.905
Dyslipidemia	83 (44%)	78 (50%)	161 (46.5%)	0.305
Baseline mRS	0.9 ± 1.4	1.3 ± 1.6	1.1 ± 1.5	**0.03**
Baseline mRS >2 (N)	36 (19%)	37 (23.6%)	73 (22%)	0.209
Admission NIHSS (mean ± SD)	15 ± 7	16 ± 7	15 ± 7	0.062
Admission ASPECTS (mean ± SD)	8.3 ± 1.2	7.5 ± 1.6		**0.02**
Admission ASPECTS admission >7	124 (84%)	102 (77%)	226 (80%)	0.135
Admission collateral score (mean ± SD)	2.1 ± 0.7	1.7 ± 0.8		**0.04**
Favorable (2–3) admission collateral score (*N*)	99 (60%)	78 (56%)	186 (48%)	0.412

Values represent a number of patients unless otherwise stated. Statistically significant p-values are marked in bold.

**Table 2:** Multivariate analysis for predictors of TVS.

**Variable**	**Odds ratio**	**95% confidence interval**	***P*-Value**
Age (per year increase)	0.98	0.96–0.99	**0.046**
mRS upon admission (per unit increase)	0.9	0.76–1.07	0.22
Collateral score (per unit increase)	1.34	1.08–1.68	**0.008**

Bold values represent statistically significant differences between the two groups.

### Recanalization treatment

The rates of treatment with intravenous tissue plasminogen activator (IV-tPA) prior to EVT were similar between the two groups (Table 3). The rates of achieving TICI 2b-3 on the first thrombectomy pass were higher in the positive-TVS group (64 vs. 36%, *p* = 0.01) although the rates of final target recanalization of TICI2b-3 were similar (79%). Moreover, the mean number of passes during EVT was significantly lower in the positive TVS vs. the negative TVS group (2.65 ± 4.6 vs. 2.93 ± 3.7, respectively, *p* = 0.03) and fewer patients required ≥3 passes to achieve recanalization in the positive TVS (30 vs. 42%, *p* = 0.04).

**Table 3:** Recanalization procedures information and clinical outcome.

**Variable**	**Positive TVS** ***N* = 189 (55%)**	**Negative TVS** ***N* = 157 (45%)**	***p*-value**
IV-tPA treatment	64 (34%)	62 (39%)	0.305
**Endovascular treatment**
First pass success	68 (51%)	37 (35%)	**0.01**
Number of passes ≥ 3	40 (30%)	44 (42%)	**0.04**
Mean number of passes	2.65 ± 4.6	2.93 ± 3.7	**0.03**
Favorable TICI score (2b-3)	100/126 (79%)	71/89 (79%)	0.94
**Post-procedural CT (≤24 h)**
ASPECTS (mean ± SD)	6.9 ± 2.2	5.2 ± 2.3	**0.001**
ASPECTS >7	70 (52%)	34 (27%)	**<0.001**
Hemorrhagic transformation	17 (9%)	13 (8.3%)	0.81
**Clinical outcome**
Discharge NIHSS (mean ± SD)	6 ± 6	9 ± 7	**0.003**
Discharge mRS (mean ± SD)	3.4 ± 1.9	4.4 ± 1.5	**<0.00001**
Discharge mRS 0–1	36 (19%)	11 (7%)	**0.002**
Discharge mRS 0–2	56 (29.5%)	19 (12%)	**0.001**
90-day mortality	25 (13.2%)	34 (21.6%)	**0.038**

Statistically significant P-values are marked in bold.

### Outcome

Despite relatively similar baseline characteristics and similar rates of ASPECTS > 7 at admission and successful recanalization (79%) in both groups, patients with positive TVS had a higher post-procedural ASPECTS (6.9 ± 2.2 vs. 5.2 ± 2.3, *p* = 0.001) and were almost twice as likely to have a post-procedural ASPECTS > 7 (52 vs. 27%, *p* < 0.001). In addition, patients with positive TVS were discharged with lower NIHSS (6 ± 6 vs. 9 ± 7, *p* = 0.003), higher rates of mRS 0–1 (19 vs. 7%, *p* = 0.002) and 0–2 (29.5 vs. 12%, *p* = 0.001; Table 3), and lower rates of 90-day mortality (13.2 vs. 21.6%, *p* = 0.038).

In a comparison between patients with mRS 0–2 to those with mRS > 2 at discharge, there was a higher rate of patients with positive TVS who were discharged with mRS 0–2 (Table 4). In a multivariate analysis to identify patient characteristics associated with mRS 0–2 (Table 5), the presence of TVS was not an independent predictor (OR 2.51, *p* = 0.13) but showed a remarkable tendency.

**Table 4:** Characteristics of patients with and without mRS 0–2 upon discharge.

	**mRS 0–2** ***N* = 75**	**mRS > 2** ***N* = 271**	***P***
Age (mean)	61.12 ± 14.51	70.79 ± 13.71	0.001
Sex (male)	41 (55%)	104 (38%)	0.13
AF	19 (25%)	142 (52%)	**0.004**
HTN	41 (55%)	188 (69%)	0.11
DM	19 (25%)	92 (34%)	0.23
DYSLIP	28 (38%)	133 (49%)	0.44
Admission mRS (mean ± SD)	0.46 ± 1.18	1.4 ± 1.6	**0.00001**
Admission NIHSS (mean ± SD)	6.72 ± 4.93	14.61 ± 5.71	**0.001**
Admission ASPECTS (mean ± SD)	8.97 ± 1.03	8.14 ± 1.34	**0.001**
Admission collateral score (mean ± SD)	2.16 ± 1.19	1.01 ± 0.93	**0.001**
Admission collateral score (median, IQR)	2, 1–3	1, 0–2	**0.001**
Positive TVS	56 (75%)	133 (49%)	**0.002**
IV-tPA	25 (33%)	42% (114)	0.3

Bold values represent statistically significant differences between the two groups.

**Table 5:** Multivariate analysis for predictors of mRS 0–2 upon discharge.

**Variable**	**Odds ratio**	**95% confidence interval**	***P*-value**
Age (per year increase)	0.95	0.92–0.97	0.034
Admission mRS (per unit increase)	0.96	0.65–1.43	0.854
Atrial fibrillation	0.36	0.1–1.38	0.137
Admission NIHSS (per unit increase)	0.81	0.73–0.9	<0.0001
Tilted-V sign	2.51	0.76–8.3	0.13
Admission ASPECT score (per unit increase)	1.93	1.14–3.27	0.015
Collateral score (per unit increase)	1.94	1.08–3.48	0.025

## Discussion

In the current study, we described TVS, an easily identified radiological marker found in association with younger patients and a better collateral score on admission CTA. Patients with AIS who were positive for TVS required fewer EVT attempts and benefited from substantially higher rates of successful recanalization on the first thrombectomy pass. As a result, patients with TVS had better post-procedural clinical and radiological outcomes, and they also had lower rates of 90-day mortality.

We cannot fully explain the underlying physiology of TVS. One possible explanation is that TVS was formed due to incomplete LVO (ILVO) with the minimal anterograde flow. Possibly, the clot might be less organized and attached to the vessel wall, thereby providing a plausible explanation for higher rates of first-pass recanalization witnessed in patients with TVS. Another possibility is the transient fluctuation of occlusion in the M1 stem, allowing the sporadic distal filling of the M2 segments. A third possibility is the formation of TVS by retrograde flow from the enriched collateral system we have found in patients with TVS. This suggests that TVS could be a surrogate marker for better collateral vasculature, which would contribute to improved functional outcomes.

A study of the LVO AIS cohort compared patients who presented with an ILVO to those with complete large vessel occlusion (CLVO) (14). Patients with ILVO had lower median NIHSS (11 vs. 15, *p* < 0.001), better recanalization rates (95.9 vs. 78.2%, *p* < 0.005), lower median groin puncture-to-reperfusion time (30 vs. 67 min, *p* < 0.001), and more favorable outcomes at 90-days (81 vs. 29.1%, *p* < 0.001) compared to those with CLVO. Similarly, patients with TVS in our study had higher rates of successful recanalization on the first pass. However, NIHSS upon admission and rates of recanalization were similar for patients with positive and negative TVS. Therefore, we presumed that TVS cannot be regarded simply as ILVO. Younger age was an independent predictor of TVS. Perhaps, patients with TVS have more elastic cerebral vasculature with a less hermetic closure of the M1-MCA lumen. We did not encounter fluctuations in the clinical syndrome that would support the hypothesis of transient fluctuations in the M2 stem occlusion. However, an improved collateral system was the strongest predictor of a positive TVS sign in our patients, possibly supporting clinical benefits due to retrograde flow.

Working with the combination of vascular and parenchymal imaging markers among patients with LVO AIS may help to achieve better-tailored and faster patient selection, thereby minimizing futile recanalization procedures (15). The prognostic implications of distal filling of occluded large cerebral vessels due to the presence of collateral vessels were previously described in anterior circulation AIS patients treated with EVT (16). Collateral status on CTA was the strongest pre-therapeutic predictor of favorable outcomes (16). In another study of patients with M1-MCA LVO, MR-perfusion assessment of the collateral vasculature showed that the velocity of collateral filling was independently associated with the rate of recanalization after intravenous thrombolysis (17). Patients with slow collateral filling on MR perfusion had lower rates of recanalization and were at relatively high risk for hemorrhagic transformation following treatment. Not surprisingly, patients with TVS had better collateral scores in the current series, had fewer ischemic changes, and presented with better ASPECTS. Interestingly, patients with positive TVS required fewer EVT attempts to achieve target recanalization, and presumably, the interval from groin puncture to successful recanalization was shorter. This in turn could minimize the volume of brain tissue undergoing ischemic changes, as suggested by higher post-procedure ASPECTS scores in the TVS group despite similar rates of successful recanalization in patients who lacked a TVS.

Our study looked at the clinical significance of TVS in patients presenting within 6 h from symptom onset. Further studies should look at the frequency and significance of TVS in patients presenting in the delayed time window (6–24 h from symptom onset), since TVS may help to achieve better patient selection in patients presenting in the delayed time window. In studies investigating TVS in the delayed time window, perfusion images and the size of the penumbra, and core should be included in the analysis.

The limitations of this study are mainly those inherent to the retrospective analysis of our cohort of patients. Accurate time frame measurements from symptom onset to recanalization were not prospectively registered and, therefore, not included in the study. Moreover, the study does not report the 90-day mRS due to incomplete follow-up for some patients. However, the ongoing prospective consecutive nature of our data acquisition in the registry minimizes the possibility of bias. Notably, our study did not look at vascular anatomical variations and their possible impact on the outcome. Further studies should thoroughly investigate the TVS sign in light of anatomical variations such as tri-furcation or quatro-furcation of the MCA or dominant early temporal M2 branches.

In conclusion, among patients presenting with AIS within the early time window (≤ 6 h from symptom onset), a positive TVS was associated with less complicated endovascular interventions and better functional outcomes. Further prospective studies may shed some more light on radiological findings related to a better prognosis among patients presenting with AIS.

## Data availability statement

The original contributions presented in the study are included in the article/supplementary material, further inquiries can be directed to the corresponding author.

## Ethics statement

The studies involving human participants were reviewed and approved by 0149-2015 and HMO-0378-2018. Written informed consent for participation was not required for this study in accordance with the national legislation and the institutional requirements.

## Author contributions

AA: writing and original draft. AHon: writing, validation, formal analysis, and visualization. AHor: conceptualization, methodology, writing—review and editing, and supervision. EA: statistical analysis. AA, AHor, EA, FA, YZ, GI, YS, IM, IS, JC, RL, and AHon: data curation. All authors contributed to the article and approved the submitted version.
